# Supplementary material for: 2passtools: two-pass alignment using machine-learning-filtered splice junctions increases the accuracy of intron detection in long-read RNA sequencing
Source: Genome Biol. 2021 Mar 1;22:72. doi: 10.1186/s13059-021-02296-0 (PMC7919322; doi:10.1186/s13059-021-02296-0)
Supplement: Supplementary file 1 — Additional file 1: Fig. S1. Simulation of nanopore DRS read alignments. Fig. S2. Annotation-guided alignment improves spliced alignment of simulated reads. Fig. S3. Junction metrics can identify genuine splice junctions. Fig. S4. Machine-learned sequence information can identify genuine splice junctions. Fig. S5. Filtered two-pass alignment improves the identification and quantification of correct transcripts without a reference annotation. Fig. S6. Filtered two-pass alignment improves genome-guided annotation. [file 13059_2021_2296_MOESM1_ESM.pdf]

2passtools: two-pass alignment using machine-learning-filtered splice junctions increases the accuracy of intron detection in long-read RNA sequencing

Supplemental Figures

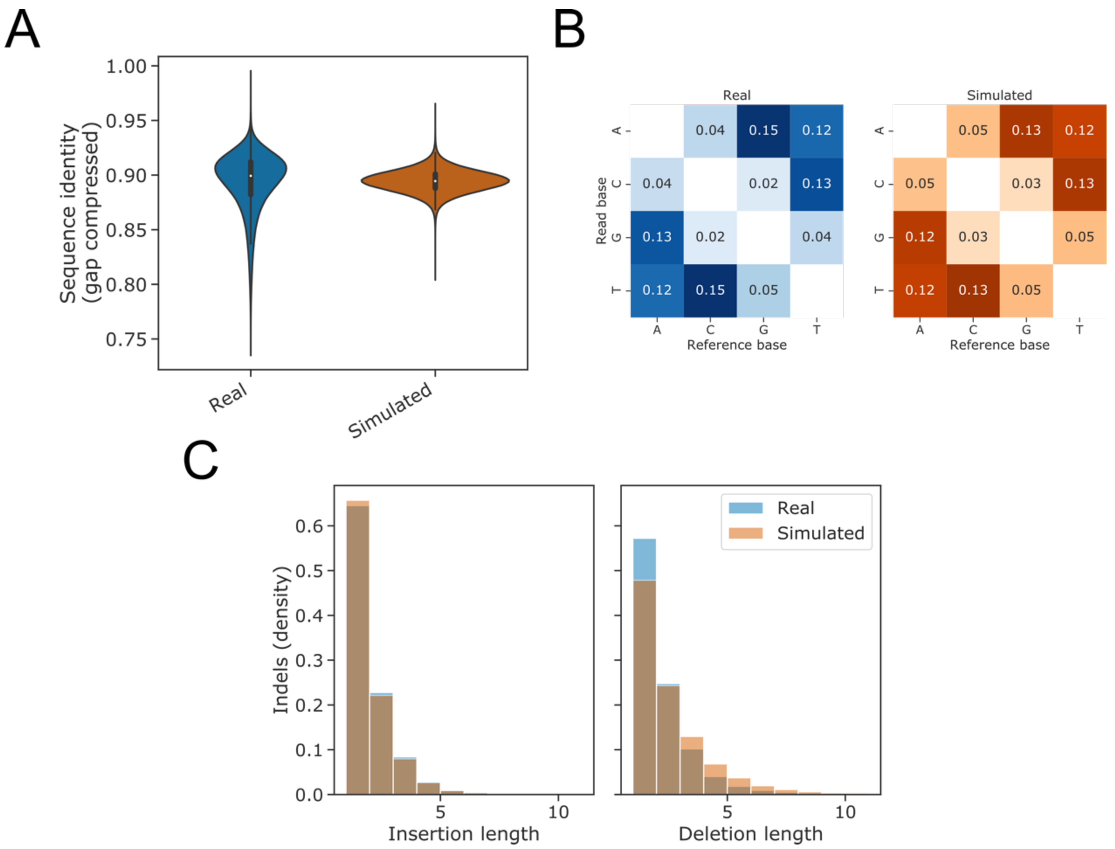

Fig. S1. Simulation of nanopore DRS read alignments. A Violin plot showing the distribution of sequence identity scores for real and simulated Arabidopsis nanopore DRS reads. Simulated reads match the median sequence identity of real reads, although they do not capture the tails of high- and low-quality reads. **B** Insertion and deletion length distributions for real and simulated nanopore DRS reads. **C** Mismatch profiles for real and simulated nanopore DRS reads.

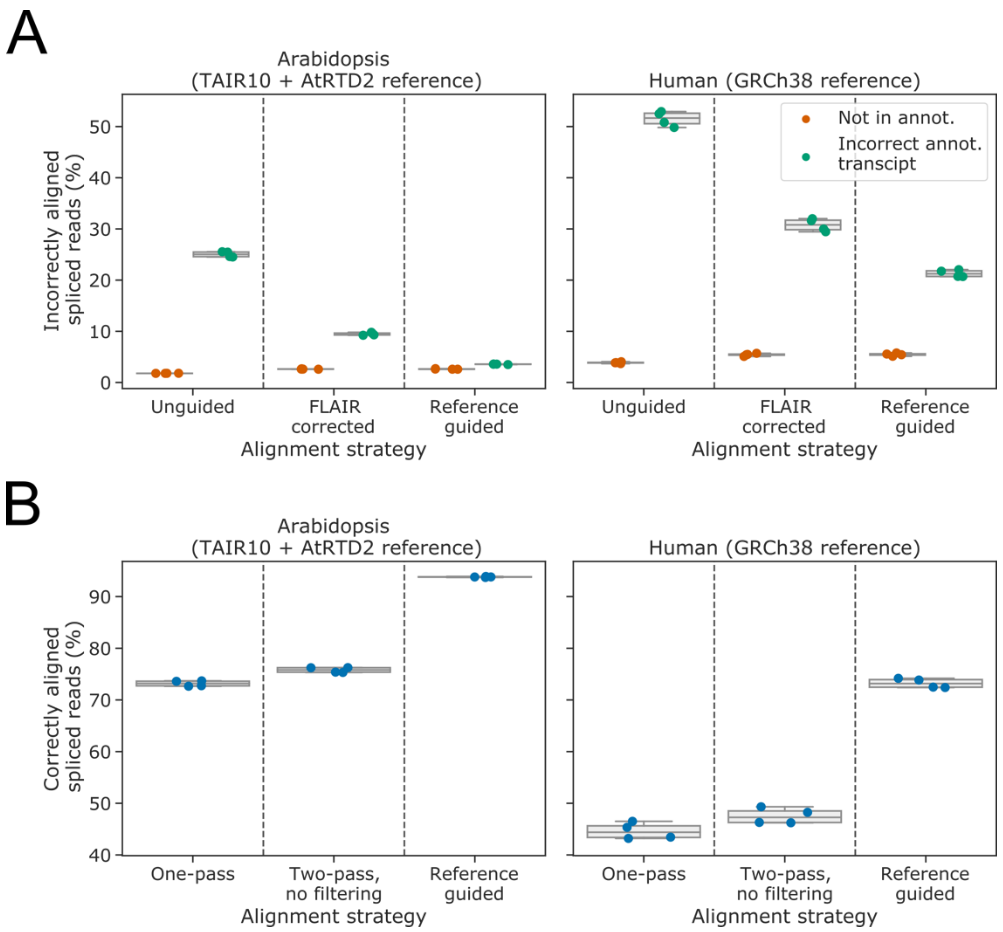

Fig. S2. Annotation-guided alignment improves spliced alignment of simulated reads. A Boxplots with overlaid strip-plot showing the percentage of alignments which do not map correctly to the splice junctions of the transcript from which they were simulated, for one-pass unguided minimap2 alignments, FLAIR-corrected alignments and reference annotation-guided minimap2 alignments. Reads that align to unannotated splice junctions or combinations of junctions ("Not in annot.") are shown in orange. Reads which align to the incorrect annotated combination of splice junctions are shown in green. Reads were simulated from Arabidopsis (left) and human (right) nanopore DRS data aligned to the AtRTD2 and GRCh38 reference transcriptomes, respectively. **B** Boxplots with overlaid strip-plot showing the percentage of alignments which map correctly to the splice junctions of the transcript from which they were simulated, for one-pass unguided minimap2 alignments, two-pass minimap2 alignment and reference annotation-guided minimap2 alignments. Reads were simulated from Arabidopsis (left) and human (right) nanopore DRS data aligned to the AtRTD2 and GRCh38 reference transcriptomes, respectively.

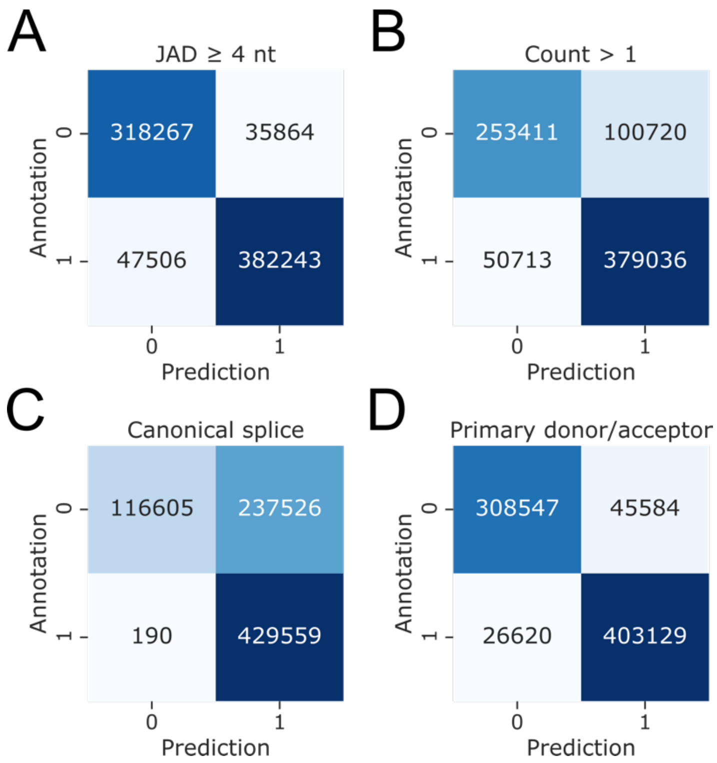

Fig. S3. Junction metrics can identify genuine splice junctions. A-D Confusion matrices showing the ratios of correct and incorrect predictions using: **A** a JAD threshold of 4 nt; **B** a count threshold of 1 nt; **C** the presence of a canonical U2 GU/AG, U12 GC/AG or U12 AU/AG intron motif; and **D** the primary donor/acceptor metric, defined as whether there are no alternate donor or acceptor sites with greater support (i.e. higher count or JAD) within 20 nt.

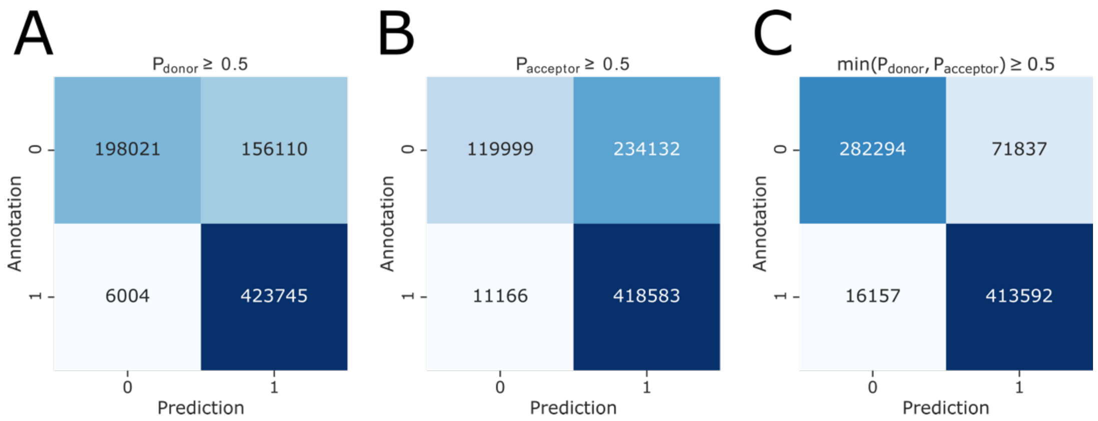

Fig. S4. Machine-learned sequence information can identify genuine splice junctions. A-C Confusion matrices showing the ratios of correct and incorrect predictions using: **A** an LR prediction threshold of 0.5 for splice site strength predictions made on donor site sequences; **B** an LR prediction threshold of 0.5 for splice site strength predictions made on acceptor site sequences; **C** a minimum prediction threshold of 0.5 for both splice donor and acceptor site sequences.

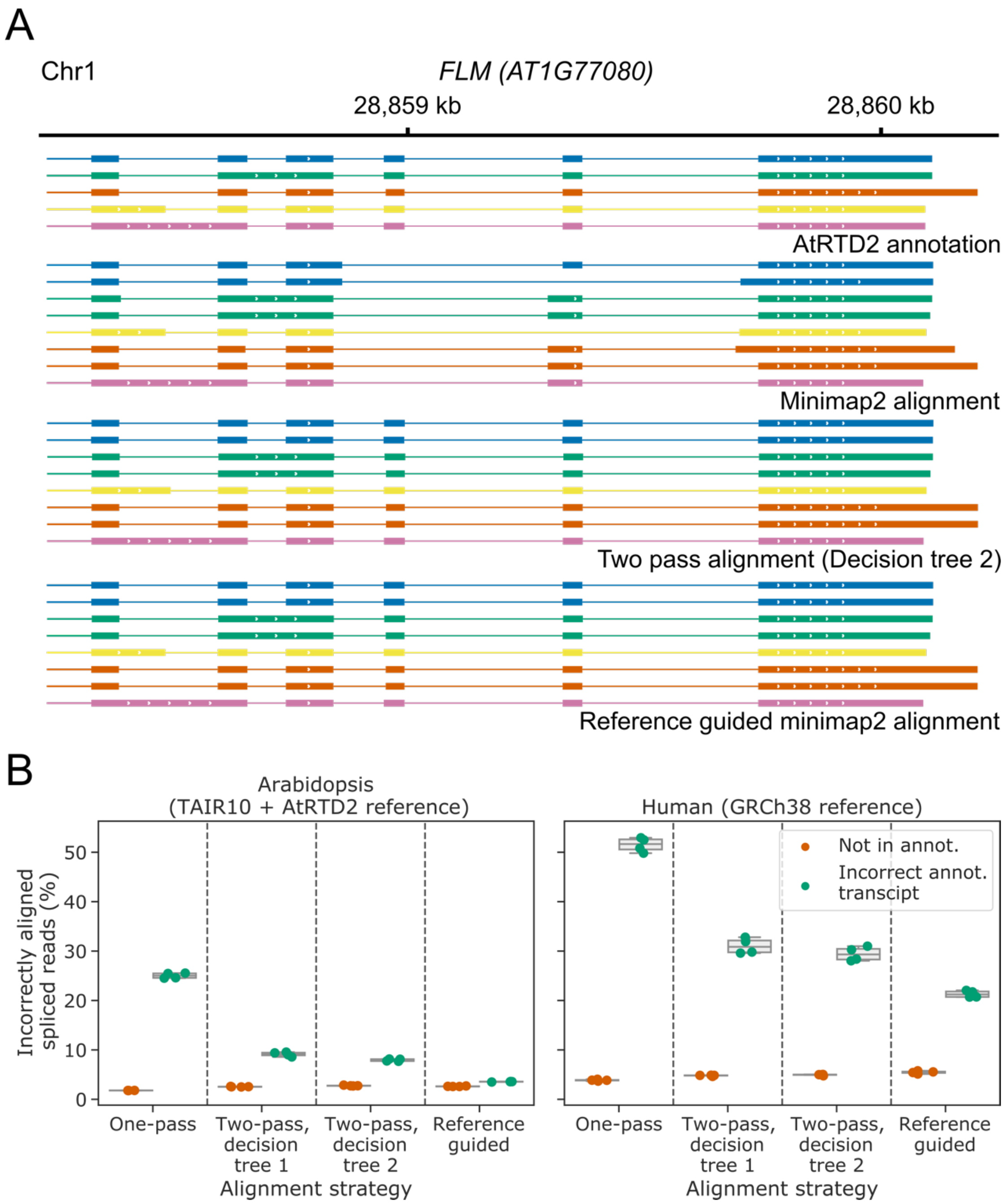

Fig. S5. Filtered two-pass alignment improves the identification and quantification of correct transcripts without a reference annotation. A Gene track showing alignment of a sample of simulated nanopore DRS reads at the Arabidopsis *FLM* gene. The AtRTD2 reference annotation, from which reads were simulated, is shown on top, with unguided minimap2 alignments, two-pass minimap2 alignments using the second decision tree classification, and reference-annotation-guided alignments shown below. Only reads where exon 6 failed to align in the initial unguided alignment are shown. Each read alignment is coloured based on the reference transcript it was simulated from, and reads are in the same order within each alignment method group. Mismatches and indels are not shown. **B** Boxplots with overlaid

strip-plots showing the percentage of alignments which do not map correctly to the splice junctions of the transcript from which they were simulated, for one-pass unguided minimap2 alignments, two-pass alignment with decision trees one and two, and reference annotation-guided minimap2 alignments. Reads that align to unannotated splice junctions or combinations of junctions are shown in orange. Reads which align to annotated combinations of splice junctions which they were not simulated from are shown in green. Reads were simulated from Arabidopsis (left) and human (right) nanopore DRS data aligned to the AtRTD2 and GRCh38 reference transcriptomes, respectively.

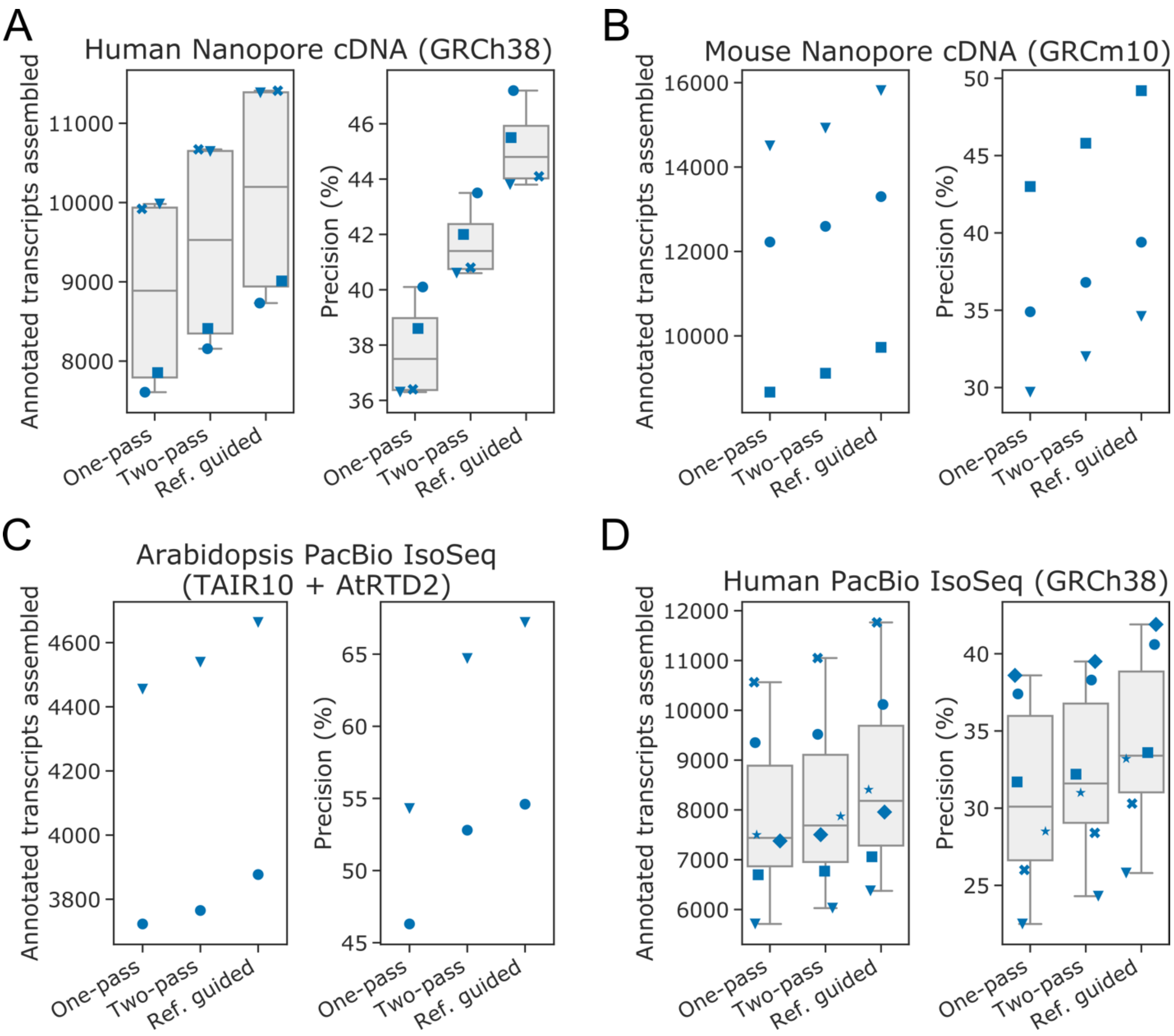

Fig. S6. Filtered two-pass alignment improves genome-guided annotation. A–D Stripplots with box-and-whiskers showing the number of correct transcripts assembled (left panels) and precision of transcripts assembled (right panels) for genome-guided transcriptome assembly using StringTie2. Two-pass alignment improved the precision and number of transcripts assembled from **A** human nanopore cDNA; **B** mouse nanopore cDNA; **C** Arabidopsis PacBio IsoSeq; and **D** human PacBio IsoSeq data. For all boxplots, overlaid strip-plots are shown for individual samples. Each sample was assigned a unique marker so that changes in the metrics could be tracked between the one-pass, two-pass and reference-guided alignments. Box-and-whiskers not shown for samples with less than 4 data points.
