# Supplementary material for: 2passtools: two-pass alignment using machine-learning-filtered splice junctions increases the accuracy of intron detection in long-read RNA sequencing
Source: Genome Biol. 2021 Mar 1;22:72. doi: 10.1186/s13059-021-02296-0 (PMC7919322; doi:10.1186/s13059-021-02296-0)
Supplement: Supplementary file 2 — Additional file 2. Review History. [file 13059_2021_2296_MOESM2_ESM.docx]

**1^st^ round**

**Reviewer 1**

This manuscript addresses the problem of improving long-reads splicing alignment, a challenging and essential problem in long-reads RNA-seq analysis, as long reads suffers from high-error rate. The proposed tool, 2passtools, has been illustrated achieving significant improvement (w/o using reference annotations). The machine-learning model is simple but efficient, with explainable features. Overall this manuscript is very well written, with all conclusions clearly stated and well-supported. Besides, insights about existing tools and techniques are also discussed, which gives practical guidance to the community for long-reads RNA-seq analysis.

I have two main concerns:

1, can 2passtools improve splicing alignment in the presence of reference annotations? It's clear (shown in this manuscript) that minimap2 (w/ annotations) can achieve better alignment then minimap2 (w/o annotations) + 2passtools. This limits the impact of proposed tool in the case of well-annotated species like human. Possible experiments: (1), align long-reads using minimap2 with annotations, and then run 2passtools: does the resulting (spliced) alignment still get improved? (2), do the same as in (1), but compare if the assembled transcripts gets improved.

2, (strongly related with 1): can 2passtools make use of the annotations to get further improved splicing alignments?

**Reviewer 2**

The manuscript by Parker, M., et al. details a new software package "2passtools" that was developed to correct splice site junctions in long error prone direct RNA sequencing reads. At its core "2passtools" uses a decision tree model that incorporates junction alignment distance, canonical splicing motifs, and a logistic regression model trained on splice site sequences. The authors highlight the importance and need of splice site correction tools, particularly in error prone long read sequencing. The results from their "2passtools" software do offer a marked improvement over unguided single pass alignment using minimap2. However, it is this reviewer's opinion that the authors failed to show significant improvement over previously published correction techniques employed by tools like FLAIR. Additionally, the manuscript leans heavily on the use of simulated reads but offers little to no results and validation applying real long-read sequencing data and use-cases. It is for these reasons and those listed below that this reviewer cannot recommend for publication without significant revisions.

Major Concerns:

1. The authors should directly compare FLAIR and StringTie2 to their final model on the same graph. Based on Figures 2b (FLAIR) and 5A (2passtools) both software show similar correctly aligned spliced read values for both Arabidopsis (~87%) and Human (~65%). A direct comparison to StringTie2 is not show anywhere in the manuscript. If the performance of the new tool does not exceed that of existing tools, the novel contribution of this tool to the field remains unclear.

2. The manuscript also lacks experimental validation. The only experimental data is compared to known annotation sites. However, as the authors point out, the strength of long read sequencing is discovering new junctions and annotating them in context of the full read for novel isoform identification and quantification. The authors should attempt to discover new sites with their software to demonstrate its use and validate those sites with either short read sequencing or PCR and sanger sequencing experiments. In addition, the tool should be validated using long-read sequencing data as well because simulated data can never truly recapitulate real data.

Minor Concerns:

1. It would be nice if the authors described their reasoning for choosing the models they used (decision tree and LR) compared to alternative machine learning models.

2. Figures that plot percent on an axis should either scale from 0 to 100% or have a defined value for each figure so that the reader can easily compare between samples. For example, in Fig 6 B-D the y-axis minimum, precision (%) values, are 66, 42.5, 47.5, and 74, respectively.

3. Additionally, box plots are not appropriate for Figures 6D, 6E, s6B, and s6C as the plots contain 3 or less data points and the statistics of a box plot are not applicable. Perhaps a line plot would be better suited for these and related figures.

4. The authors should explain the reasoning for altering default minimap2 splicing parameters in their methods. Without the clarification, it is unclear whether the authors find that these parameters generally help correct splice site identification or if the settings were chosen to favor their method.

**Author response**

**Reviewer #1:**

*This manuscript addresses the problem of improving long-reads splicing alignment, a challenging and  essential problem in long-reads RNA-seq analysis, as long reads suffers from high-error rate. The  proposed tool, 2passtools, has been illustrated achieving significant improvement (w/o using  reference annotations). The machine-learning model is simple but efficient, with explainable  features. Overall this manuscript is very well written, with all conclusions clearly stated and well supported. Besides, insights about existing tools and techniques are also discussed, which gives practical guidance to the community for long-reads RNA-seq analysis.*

• We thank the reviewer for these kind remarks.

*I have two main concerns:*

*1, can 2passtools improve splicing alignment in the presence of reference annotations? It's clear  (shown in this manuscript) that minimap2 (w/ annotations) can achieve better alignment then  minimap2 (w/o annotations) + 2passtools. This limits the impact of proposed tool in the case of well annotated species like human.*

• We anticipate that the main impact of 2passtools will be in newly sequenced species where  there are currently little or no annotations. There are a growing number of such projects  that include the African Orphan Crops Consortium, which we are part of; the Darwin tree of  life project; and the Earth BioGenome Project, which aims to sequence all eukaryotic life on  Earth. We state this in our manuscript and have clarified this further in the new section  *“Two-pass alignment can aid novel splice-isoform discovery in annotated species”* of the  revised version.

• That said, we recognise that identifying novel splice isoforms in well annotated species  remains an important and recurring research question, because different conditions,  genotypes, or increased sequencing depth continue to reveal new splice variants. We have  therefore addressed whether 2passtools can improve spliced alignment in well-annotated  species, in two new Results & Discussion sections entitled *“Two-pass alignment can aid novel  splice-isoform discovery in annotated species”* and “*Two-pass alignment discovers novel splice*

*isoforms in the Arabidopsis RNA exosome mutant hen2-2”.* We explain the detail of these  sections below.

*Possible experiments: (1), align long-reads using minimap2 with annotations, and then run  2passtools: does the resulting (spliced) alignment still get improved? (2), do the same as in (1), but  compare if the assembled transcripts gets improved.*

*2, (strongly related with 1): can 2passtools make use of the annotations to get further improved  splicing alignments?*

• We thank the reviewer for these experiment suggestions. We decided to address questions  (1) and (2) together. To do this, we created a new reference-aided mode for 2passtools,  which is explained in the first paragraph of the new Results & Discussion section “*Two-pass  alignment can aid novel splice-isoform discovery in annotated species*”. In this mode, reads  should first be aligned using reference-guided minimap alignment. Annotated splice  junctions are then passed to 2passtools along with alignments and used as labels to train  random forest models which can classify novel genuine splice junctions. These can then be  added to the annotated splice junctions and used to perform second-pass alignment.

• For simulated data, the reference annotation represents the perfect ground truth – there  are no false negative or false positive introns in the dataset. It follows that using 2passtools after reference-guided alignment cannot improve read-alignments, as it can only introduce  false positives - there are no false negatives not present in the reference to rescue.

• In reality, all reference annotations are incomplete – even for the human genome, new RNA  sequencing studies continue to identify previously unannotated alternatively processed  mRNAs and lncRNAs, indicating that there are further splice isoforms yet to be discovered^2^.

• For each genome there is expected to be some level of reference “completeness” where  false positive introns introduced by 2passtools begin to outweigh false negatives rescued. We therefore performed a “reference missingness” experiment where we randomly  removed varying percentages of transcript isoforms from the reference annotation, and  performed annotation aided two-pass alignment.

• We find that in well annotated conditions (<10% missingness), 2passtools correctly identifies  novel splice junctions with high recall, and can improve overall alignment. We have added a  new text section to the Results & Discussion to explain this new analysis (“*Two-pass  alignment can aid novel splice-isoform discovery in annotated species*”), with the  corresponding data analysis presented in Figure 7.

• Whilst it is hard to judge the missingness of any real genome annotation, this section  provides evidence-based guidance on when to use 2passtools, either with or without  reference annotations.

**Reviewer #2:**

*The manuscript by Parker, M., et al. details a new software package "2passtools" that was developed  to correct splice site junctions in long error prone direct RNA sequencing reads. At its core  “2passtools" uses a decision tree model that incorporates junction alignment distance, canonical  splicing motifs, and a logistic regression model trained on splice site sequences. The authors highlight  the importance and need of splice site correction tools, particularly in error prone long read  sequencing.*

• We again thank the reviewer for these comments.

*The results from their "2passtools" software do offer a marked improvement over unguided single  pass alignment using minimap2. However, it is this reviewer's opinion that the authors failed to show  significant improvement over previously published correction techniques employed by tools like  FLAIR.*

• Whilst FLAIR uses splice junctions from an existing reference annotation to correct read  alignments, 2passtools seeks to correct them using no orthogonal information. This is critical  when high-quality reference annotations do not already exist – for example in newly  sequenced organisms, or mutants/conditions with altered gene expression and patterns of  splicing.

• As the reviewer notes, 2passtools does offer a “marked improvement” over unguided single  pass alignment using minimap2, which is the main use case for 2passtools – i.e. when  reference annotations are unavailable. In this instance it would not be possible to use FLAIR.

• Furthermore, despite the fact that FLAIR is supplied with the “ground truth” set of splice  junctions and 2passtools is not, we find that 2passtools still achieves a higher percentage of  correctly aligned reads than FLAIR. We argue that this also represents an improvement given  the difference in inputs.

*Additionally, the manuscript leans heavily on the use of simulated reads but offers little to no results  and validation applying real long-read sequencing data and use-cases.*

• **Genome Biology** guidelines for methods or software papers state that “where possible, the  method should be benchmarked using a synthetic dataset (or other dataset where the  ground truth is known)”. Consistent with this, we have used synthetic data for the  benchmarking analysis. The performance of the tool was also evaluated on experimental  data in the transcriptome assembly section, where it was applied to real datasets from  Arabidopsis, human, mouse and yeast (See Figures 6B-E, Figure S6).

• In this revised version of the manuscript we have added validation from a mutant with  altered RNA processing and splicing. We analysed the Arabidopsis RNA exosome mutant  *hen2-2* and detail our findings in a new Results & Discussion section *“Two-pass alignment*

*discovers novel splice isoforms in the Arabidopsis RNA exosome mutant hen2-2*”. Further  detail on this section is given below.

*1. The authors should directly compare FLAIR and StringTie2 to their final model on the same graph.*

• The use-cases of FLAIR and 2passtools are different because FLAIR requires high-confidence  splice junctions to be provided to correct reads, whilst 2passtools attempts to learn them *de  novo*. These tools therefore have different inputs and use cases and so a direct comparison  is not appropriate. We explain this point in the revised section “*Alignment metrics enable  identification of genuine splice junctions*”.

• Of the two methods that we explore for aligning reads when an annotation is available  (FLAIR and reference-guided minimap2), we find that reference-guided minimap2 performs  better – reference-guided minimap2 aligned a median of 5.9% and 9.6% more reads  correctly than FLAIR in simulated Arabidopsis and human datasets, respectively.

• Since reference-guided minimap2 alignment is the (current) gold-standard for alignment using an existing reference annotation, we only compare 2passtools to this method in later  sections. However, it is important to note that we are not comparing reference-guided  alignment to 2passtools to decide which is better. Instead, reference-guided alignment represents a positive control – i.e. the best possible performance that two-pass alignment  could achieve by perfectly classifying all genuine and spurious splice junctions from the first pass alignment.

• We have added text to the Results & Discussion sections "*Alignment metrics enable  identification of genuine splice junctions"* and "*Two-pass alignment with filtered splice  junctions improves transcript identification"* explaining that this decision was taken, and that  the comparison is not to benchmark relative performance but to serve as a positive control.

• StringTie2 is not a read-alignment correction tool, but a transcript assembly tool. It therefore  follows that we cannot compare 2passtools to StringTie2 – indeed in generating figure 6, the  tools are both used in serial. To clarify this point, we have added an explanation of the use  cases for StringTie2 to the revised Introduction section.

*Based on Figures 2b (FLAIR) and 5A (2passtools) both software show similar correctly aligned spliced  read values for both Arabidopsis (~87%) and Human (~65%).*

• This point is addressed above, but to clarify again, FLAIR requires annotated junctions to  guide alignment, whilst 2passtools does not. Despite this, 2passtools performs better than  FLAIR in terms of percentage of correctly aligned reads for both Arabidopsis and human  datasets.

*A direct comparison to StringTie2 is not show anywhere in the manuscript.*

• This point is also addressed above. To be clear, we cannot compare 2passtools to StringTie2  directly because 2passtools is a read-alignment correction tool, whilst StringTie2 is a genome-guided transcript assembly tool.

*If the performance of the new tool does not exceed that of existing tools, the novel contribution of  this tool to the field remains unclear.*

• To our knowledge, there are currently no other tools that can improve the single-read  spliced alignment of long reads without requiring orthogonal information from a reference  annotation or short read sequencing dataset. This is one of the key contributions of  2passtools.

*2. The manuscript also lacks experimental validation. The only experimental data is compared to  known annotation sites. However, as the authors point out, the strength of long read sequencing is  discovering new junctions and annotating them in context of the full read for novel isoform  identification and quantification. The authors should attempt to discover new sites with their  software to demonstrate its use and validate those sites with either short read sequencing or PCR  and sanger sequencing experiments. In addition, the tool should be validated using long-read  sequencing data as well because simulated data can never truly recapitulate real data.*

• To address this point on experimental validation, we have included a new case study of the  Arabidopsis RNA exosome mutant *hen2-2* in our revised manuscript. The RNA exosome  functions in RNA processing and surveillance, promoting RNA decay^3^. As a result, many of its  targets are detected at relatively low levels in wild-type backgrounds and consequently are  poorly characterised and annotated^1,3^. Therefore, sequencing of *hen2-2* mutants reveals a  rich diversity of novel transcripts^1^. We have performed transcriptome-wide sequencing of  the *hen2-2* mutant by nanopore DRS together with a deep, highly replicated Illumina RNA Seq dataset that we recently published in eLife^1^.

• Using this combination of new data, we show that of the novel splice junctions captured by  nanopore DRS which have support in *hen2-2* Illumina RNAseq data, 67% are identified as  genuine by 2passtools. We conclude that 2passtools can be used to identify genuine novel  splice junctions obtained from well-annotated organisms in less-well annotated conditions.  The associated text describing this work is found in the new section “*Two-pass alignment  discovers novel splice isoforms in the Arabidopsis RNA exosome mutant hen2-2*” in Results & Discussion and in Figure 8.

*1. It would be nice if the authors described their reasoning for choosing the models they used  (decision tree and LR) compared to alternative machine learning models*

• We chose to use decision tree models because these are human-interpretable and can be  pruned to prevent overfitting.

• Logistic regression models were chosen over random forests for the splice sequence  strength predictions because we found that they performed similarly, indicating that there  were few important interactions between sequence features.

• We have added explanations for these choices to the Results & Discussion sections entitled:  "*Alignment metrics enable identification of genuine splice junctions*" and "*A combination of  splice junction alignment metrics and sequence information...*".

*2. Figures that plot percent on an axis should either scale from 0 to 100% or have a defined value for  each figure so that the reader can easily compare between samples. For example, in Fig 6 B-D the y axis minimum, precision (%) values, are 66, 42.5, 47.5, and 74, respectively.*

• We do not agree that the results in Fig 6 B-D need to be on the same scale, since they are  obtained from different organisms and using different sequencing technologies.  Consequently, we would not suggest comparing between figures. Instead, readers need to  be able to compare the three approaches used within each figure. This would be more difficult if the figures were scaled from 0 to 100%.

• To clarify this point, we have added the following text to the corresponding revised figure  legends of Fig 6 B-D: “*Y limits vary between figures since within-figure (i.e. same species and  sequencing technology) comparison is more important than between-figure comparisons”.*

*3. Additionally, box plots are not appropriate for Figures 6D, 6E, s6B, and s6C as the plots contain 3  or less data points and the statistics of a box plot are not applicable. Perhaps a line plot would be  better suited for these and related figures.*

• We agree with the reviewer and we have removed box and whiskers from any figure with  three or less datapoints to make strip-plots. As a result, we have changed the presentation  of Figures 6D, and S6B-C. We have added text explaining this decision to the figure legends.

4. The authors should explain the reasoning for altering default minimap2 splicing parameters in  their methods. Without the clarification, it is unclear whether the authors find that these  parameters generally help correct splice site identification or if the settings were chosen to favor  their method.

• We apologise for the confusion. Most of the stated parameters (apart from the --junc-bonus  parameter and maximum intron size) are equivalent to the spliced alignment preset of  minimap2. We have revised the Methods section to make this clear.

• Maximum intron sizes reflect the intron length distributions from the organisms studied. We  have added text to clarify this to the corresponding Methods section.

• Junction bonus was increased from 9 to 12 because we found that this improved the  percentage of correctly aligned reads when performing reference-guided alignment. We  have added text explaining our choice of junction bonus to the revised corresponding Methods section.

**References**

• 1 Parker, M. T. *et al.* Nanopore direct RNA sequencing maps the complexity of  Arabidopsis mRNA processing and m6A modification. *eLife* **9**, doi:10.7554/eLife.49658  (2020).

• 2 Deveson, I. W. *et al.* Universal Alternative Splicing of Noncoding Exons. *Cell Systems* **6**, 245-255.e245, doi:10.1016/j.cels.2017.12.005 (2018).

• 3 Chen, X. *et al.* The RNA Helicases AtMTR4 and HEN2 Target Specific Subsets of  Nuclear Transcripts for Degradation by the Nuclear Exosome in Arabidopsis thaliana. *PLoS  Genetics* **10**, doi:10.1371/journal.pgen.1004564 (2014).

**2^nd^ round**

**Reviewer 1**

I appreciate the authors' efforts in addressing the reviewers' comments, and I found that they were addressed adequately. I am also convinced that 2passtools will be a useful tool for long-reads RNA-seq analysis.
